# Effects of ownership patterns on cross-boundary wildfires

**DOI:** 10.1038/s41598-021-98730-1

**Published:** 2021-09-29

**Authors:** Ana M. G. Barros, Michelle A. Day, Thomas A. Spies, Alan A. Ager

**Affiliations:** 1 College of Forestry, Oregon State University, Corvallis, OR USA; 2 Missoula Fire Sciences Laboratory, USDA Forest Service, Rocky Mountain Research Station, Missoula, MT USA; 3 USDA Forest Service, Pacific Northwest Research Station, Corvallis, OR USA

**Keywords:** Ecology, Environmental sciences, Environmental social sciences, Natural hazards

## Abstract

Understanding ownership effects on large wildfires is a precursor to the development of risk governance strategies that better protect people and property and restore fire-adapted ecosystems. We analyzed wildfire events in the Pacific Northwest from 1984 to 2018 to explore how area burned responded to ownership, asking whether particular ownerships burned disproportionately more or less, and whether these patterns varied by forest and grass/shrub vegetation types. While many individual fires showed indifference to property lines, taken as a whole, we found patterns of disproportionate burning for both forest and grass/shrub fires. We found that forest fires avoided ownerships with a concentration of highly valued resources—burning less than expected in managed US Forest Service forested lands, private non-industrial, private industrial, and state lands—suggesting the enforcement of strong fire protection policies. US Forest Service wilderness was the only ownership classification that burned more than expected which may result from the management of natural ignitions for resource objectives, its remoteness or both. Results from this study are relevant to inform perspectives on land management among public and private entities, which may share boundaries but not fire management goals, and support effective cross-boundary collaboration and shared stewardship across all-lands.

## Introduction

Human activity is a major driver of landscape change, and its impacts often manifest through patterns of ownership and respective land management practices. Differences in human values and land-use practices create mosaics of land cover^1^ and shape spatial patterns of disturbance regimes^2–4^. Humans exert bottom-up pressure on fire regimes by altering vegetation, and igniting and suppressing fires. Top-down human influences include society's changing values for forests and wildfire that affect land and fire management policies, and anthropogenic climate change leading to increasingly severe fire seasons^5,6^. Broad ownership categories can serve as a surrogate for forest management goals, practices, and policy preferences by individuals^7^, and much of the push and pull between humans, forests, and wildfire can be interpreted through the lens of landscape patterns of ownership, where land management regimes are expressed.

Landowners have different management goals for their lands. On public lands, management goals are determined by policy and regulatory frameworks but are also shaped by the public's stewardship values for public lands. In the western US, public wilderness and other congressionally protected areas such as national parks and designated reserves are set aside and managed for natural processes^8^. Other federal forest lands are managed for multiple ecosystem services. Federal managers rely on a variety of tools, from mechanized treatments and prescribed fire to the management of natural ignitions for resource objectives^9^, in what has been called a living with wildfire strategy^10^. Tribal lands may also be managed for multiple uses but have unique objectives associated with first foods, ceremonial, or spiritual uses, often including the use of fire as a management tool^11^. Private industrial forests are typically located in productive environments and are managed to maximize the growth and yield of wood products^12^. Industrial and state forest owners typically do not use prescribed fire (other than for post-harvest slash reduction, although with some exceptions) and aggressively suppress fire as part of what has been called a fortress protection strategy^10^. Private non-industrial forest owners typically avoid fire, may attempt to reduce fire risk through mechanical treatments of vegetation around homesites, and support strong fire suppression strategies. Private non-industrial forests are prioritized for fire suppression and fuel reduction to protect homes and lives by local, state and federal fire management agencies.

The implications of different land management policies and landowner behavior on the spread of wildfire through fragmented systems are not as well studied as wildfire itself^13,14^. Untangling the effect of land ownership and wildfire management policies on cross-boundary fire exposure is needed to develop and implement wildfire management policies that support functional risk governance systems and foster landscape resiliency^4,15,16^. Legislation that authorizes and rewards cross-boundary forest and fuel management^17,18^ could benefit from a quantitative foundation defining how existing policies compromise collective action and predispose mixed ownership landscapes to shared fire exposure. Moreover, ownership is often associated with perceived responsibility in creating and maintaining potential for unwanted wildfire based on how fuel and fires are managed (differently) across diverse ownerships. A common and data-driven understanding of how land ownership patterns affect fire exposure could help mitigate the fault-finding that sometimes characterizes public perceptions of wildfire in multi-ownership landscapes^10,16^.

In this work, we conduct an empirical analysis of historical fire events and examine how area burned is related to ownership. We examined fire perimeters from 1581 fires between 1984 and 2018 that burned within the states of Washington and Oregon in the US Pacific Northwest region. We overlaid the fire perimeters with ten major ownership classes and asked:

1. Do land ownerships with a concentration of highly-valued resources (e.g., buildings, timber) burn less than protected areas managed for ecological values?
2. Do patterns of disproportionate burning for a given ownership vary between forest and grass-shrub vegetation types?

We hypothesize that where highly valued resources are concentrated, strong fire suppression policies will result in disproportionally less area burned regardless of ownership and vegetation type (forest or grass/shrub) and that protected areas will burn more than expected given policies that promote resource objective fire. Results from this analysis broadly inform how fire interacts with different ownerships, a necessary step towards informed discourse to support the collective action that is needed to increase forest resilience and the protection of people and valued resources across all lands^16^.

## Methods

### Study area, potential vegetation, and wildfire data

**Figure 1:**
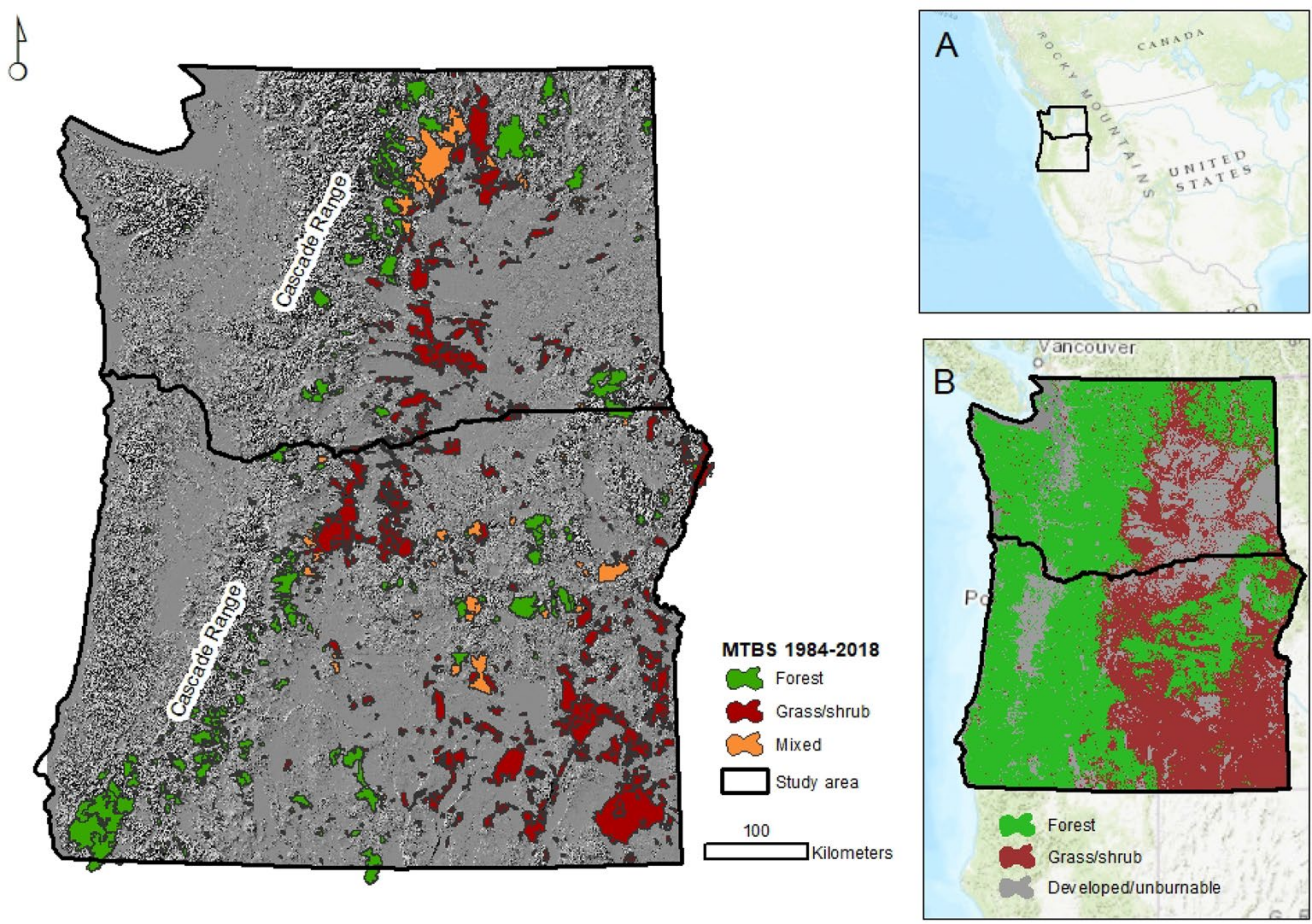
Location of the study area in western USA (inset **A**), potential vegetation types reclassified into forest and grass/shrub (inset **B**) and MTBS^22^ fire perimeters used in this analysis reclassified according to area burned in forested and burnable non-forested vegetation types. Figure produced with ArcMap 10.6.1 https://desktop.arcgis.com. Service layers credit: Esri, HERE, Garmin, Intermap, increment P Corp., GEBCO, USGS, FAO, NPS, NRCAN, GeoBase, I.G.N., Kadaster NL, Ordnance Survey, Esri Japan, METI, Esri, China (Hong Kong), © OpenStreetMap contributors and the GIS User Community.

The study area is the states of Oregon and Washington (Fig. 1, inset A) within the US Pacific Northwest region. This area contains 10.4% of the forest area in the conterminous US^19^. The region includes three distinct ecological regions: (1) dense coastal forest dominated by western hemlock (*Tsuga heterophylla*) and Sitka spruce (*Picea sitchensis*), and montane forests of Douglas-fir (*Pseudotsuga menziessii*), hemlock, and true firs (*Abies* spp.) on the west slope of the Cascade Mountains, (2) dry fire-frequent pine forest on the east side of the Cascade Mountains containing ponderosa pine (*Pinus ponderosa*), western larch (*Larix occidentalis*), lodgepole pine (*Pinus contorta*), and grand fir (*Abies grandis*)^20^ and (3) non-forested vegetation types dominated by juniper (*Juniperus* spp.) woodlands, and sage steppe.

We used potential vegetation data for Oregon, Washington, and inland Northern California^21^ to identify forested, grassland/shrubland and unburnable areas (Fig. 1, inset B). The classification resulted in 51% of the study area classified as forest and the remaining classified as grasslands and shrublands (hereafter grass/shrub) (31%), and developed/unburnable areas (18%).

We obtained fire perimeter data from the Monitoring Trends in Burn Severity (MTBS) project fire atlas, which includes fires between 1984 and 2018^22^. We selected all perimeters (with the exception of prescribed fires) with their centroid contained within the study area (*n* = 1365). We included the 2002 Biscuit fire which crossed state boundaries and extended from southern Oregon to northern California. When fire perimeters consisted of multiple disjoint polygons, we treated each polygon as an independent observation. This process resulted in 1602 individual fire footprint polygons (hereafter, fire footprint) for analysis. MTBS fire perimeters and thematic severity maps have been used by the fire science community for over a decade but have limitations, including unburned islands within the fire footprint and burned areas excluded from perimeter boundaries^23^. For the Pacific Northwest, Meddens et al.^24^ combined field observations with nonparametric classification algorithms to separate burned and unburned locations in 19 fires and found that the average unburned proportion was 20%, with high variability between fires (standard deviation = 16.4%).

**Figure 2:**
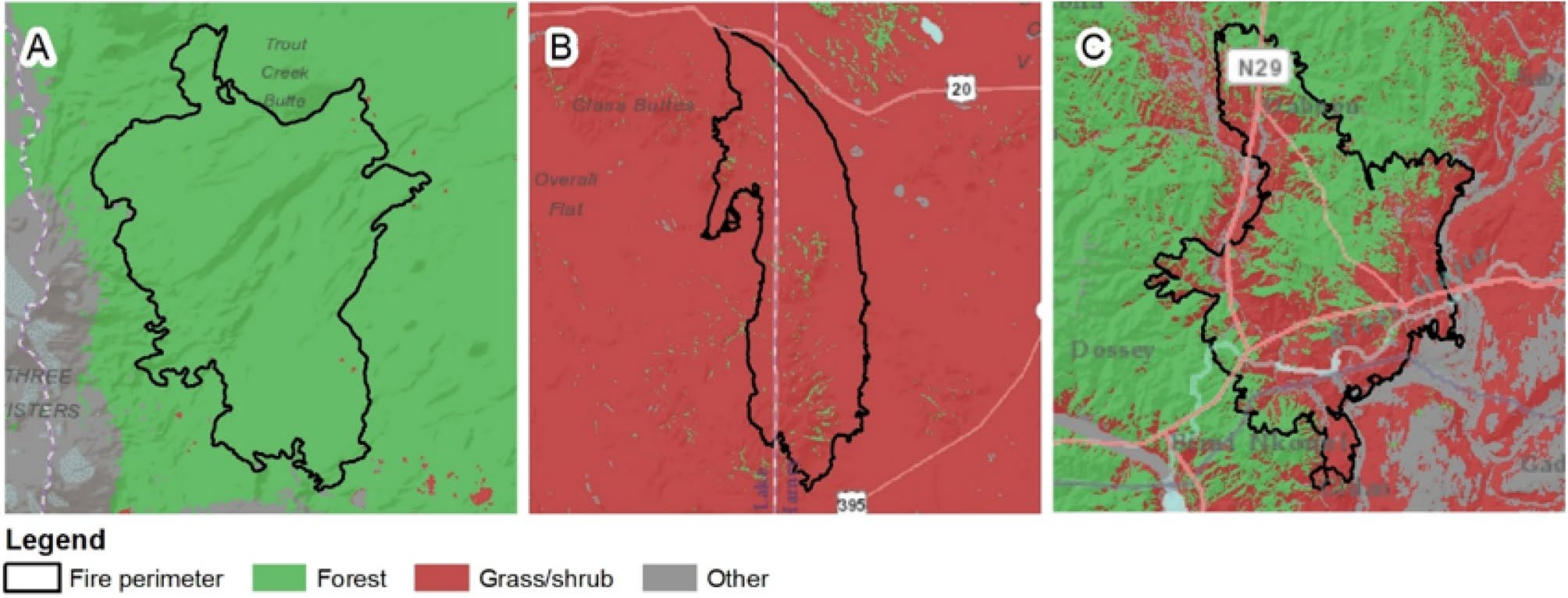
Examples of classification of three fire events into fire types based on dominant vegetation type burned. (**A**) Pole Creek Fire, OR, 2012 was classified as a forest fire, (**B**) Cinder Butte fire, OR, 2017 was classified as a grass/shrub fire and (**C**) Carlton Complex fire, WA, 2014 was classified as a mixed fire. Figure produced with ArcMap 10.6.1 https://desktop.arcgis.com. Service layers credit: Esri, HERE, Garmin, Intermap, increment P Corp., GEBCO, USGS, FAO, NPS, NRCAN, GeoBase, I.G.N., Kadaster NL, Ordnance Survey, Esri Japan, METI, Esri, China (Hong Kong), © OpenStreetMap contributors and the GIS User Community.

We classified individual fire footprints as forest, grass/shrub, and mixed fires. Assignment of vegetation class to each fire footprint was based on the proportion of vegetation type burned. When the area burned by a given event had a ratio of 2:1 (or higher) for a single vegetation type, the observation was classified according to the predominant vegetation type (Fig. 2). Footprints with ratios ≤ 2:1 were classified as mixed (Fig. 2). This assignment resulted in 484, 980, and 96 fires classified as forest, grass/shrub, and mixed fires, respectively (Fig. 1).

### Ownership

**Figure 3:**
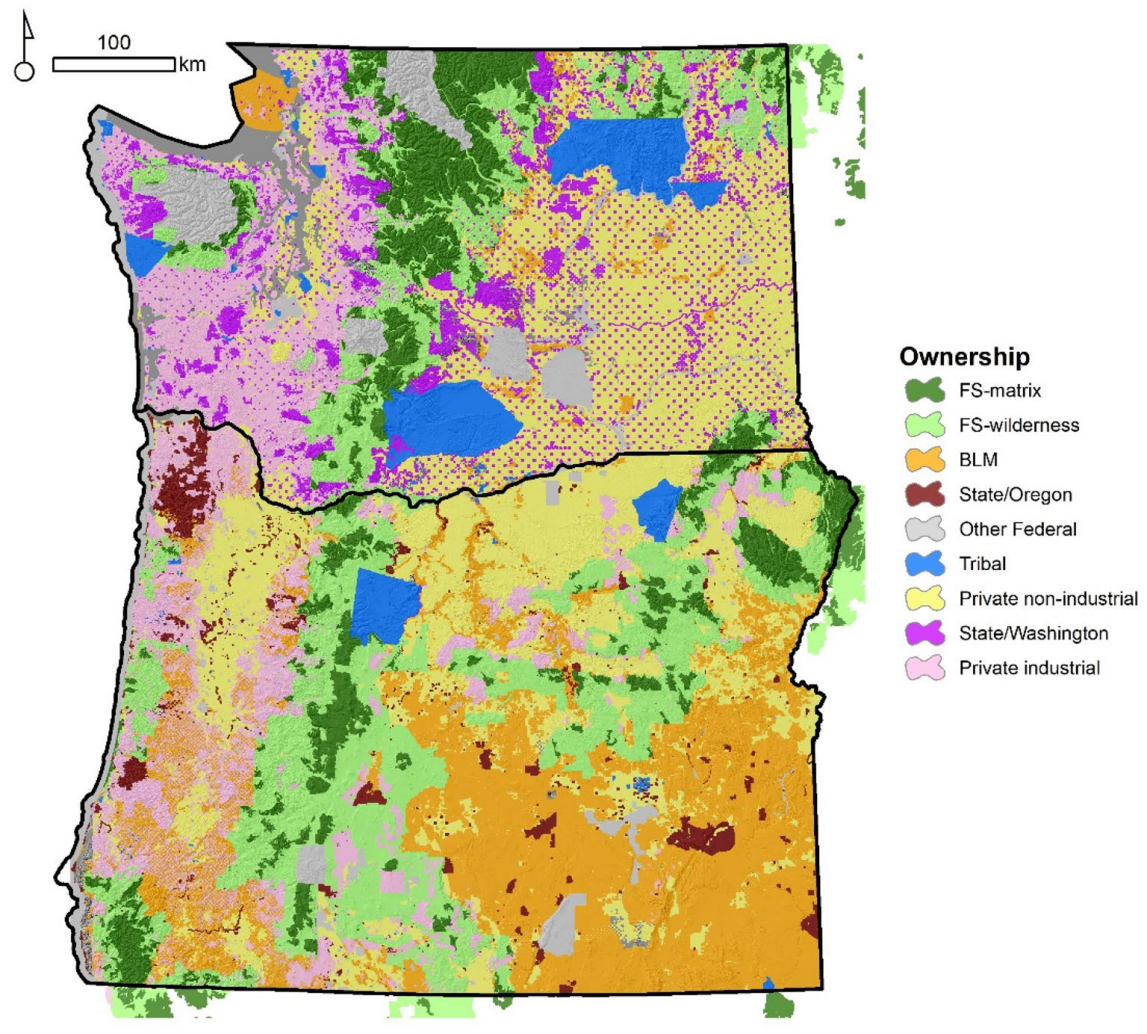
Major land ownership classes in the Pacific Northwest, USA. BLM = Bureau of Land Management, FS = United States Forest Service. State/Oregon and State/Washington denote state managed lands. Other non-federal lands not shown. Figure produced with ArcMap 10.6.1 https://desktop.arcgis.com. Service layers credit: Esri, HERE, Garmin, Intermap, increment P Corp., GEBCO, USGS, FAO, NPS, NRCAN, GeoBase, I.G.N., Kadaster NL, Ordnance Survey, Esri Japan, METI, Esri, China (Hong Kong), © OpenStreetMap contributors and the GIS User Community.

We used ownership data to identify broad ownership categories and land management policies within a category (Fig. 3). We use the term ownership as a general category that encompasses ownership and/or agency responsible for land management in that tract of land. For example, public lands are not owned but rather managed by the USDA Forest Service (FS). For simplicity, we refer to the agency as the owner.

We used the US Protected Areas Database (PAD-US) to identify FS and Bureau of Land Management (BLM) lands^25^. We further subdivided FS lands into managed (FS-matrix) and protected lands i.e., congressionally designated wilderness (FS-wilderness). These do not correspond to different ownerships but land allocations that reflect different land management policies. FS-wilderness lands are predominantly high-elevation mesic forests where historic fire regimes are characterized by infrequent high severity fire. FS-matrix lands are mostly dry and moist forest in low to mid-elevations. On the eastside of the Cascades BLM lands occupy large tracts of grass/shrub vegetation types. In Washington, BLM lands are interspersed with tracts of private non-industrial in a checkerboard pattern, whereas in Oregon, BLM lands occupy large continuous swaths of juniper woodlands and sagebrush steppe.

PAD-US was also used to identify federally recognized tribal lands, including the Confederated Tribes of the Yakama Nation, the Colville Confederated Tribes, the Kalispel Tribe, the Spokane Tribe, the Burns Paiute Tribe, the Confederated Tribes of the Umatilla Indian Reservation, and the Confederated Tribes of Warm Springs on the eastside of the Cascades. Tribal lands are distributed across low-elevation dry to mix forest and non-forested systems. The source of public state lands was the Oregon Department of Forestry for Oregon (State/Oregon) and Washington Department of Natural Resources for Washington (State/Washington). Private ownership was classified into private industrial and private non-industrial and sourced from the Integrated Landscape Assessment Project ownership layer^26^. Most state and private industrial lands consist of forested lands. Private non-industrial is a combination of forested and non-forested lands.

We mapped ownership of areas burned by the 2002 Biscuit fire in California (which burned over 200,000 ha) and surrounding areas using data from PAD-US combined with information from Google Earth and local forest and fire managers.

### Ownership selection by wildfire framed as a habitat selection problem

Wildfire has been likened to an ‘herbivore’^27^ due to its impacts on and interactions with biotic communities and ecosystems. Wildfire consumes complex organic material and converts it to organic and mineral products, unlike other natural disturbances such as hurricanes. The comparison between wildfires and herbivores is also useful from the perspective of resource selection: resource use relative to availability^28–31^.

We used a resource availability framework to determine whether different ownerships burned disproportionate to their availability^28^. If the proportion of ownership burned is less than the proportion available to burn for a specific fire footprint, then we refer to that fire as *avoiding* that particular ownership*.* Conversely, if the proportion of ownership burned is greater than the proportion available to burn, then that specific fire will be referred to as *preferring* that ownership. When proportion burned and available are equal, the fire was *indifferent* to ownership. Indifference might occur, for instance, under severe weather-driven fire events when fuel availability and suppression capacity play a minor role in fire spread and the resulting perimeter boundary.

Evaluating whether a given ownership burns disproportionally to its availability depends upon the definition of what was available to burn (resource availability) and what actually burned (resource use). This is analogous to determining the amount of habitat that is available for an animal to forage and then quantifying the types of foods available and consumed.

**Figure 4:**
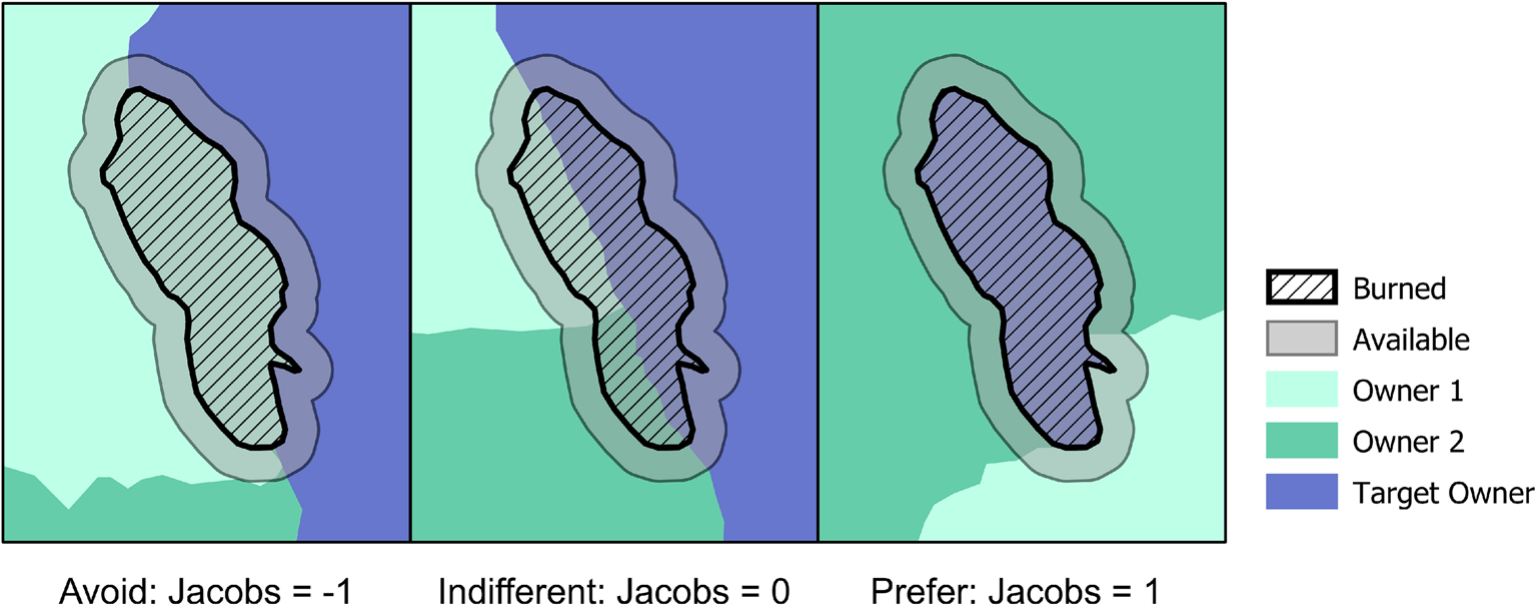
Example of the delineation of *used area* and *available area* for a fire perimeter and three alternative patterns of fire use specific to a single target owner (shown in dark blue). The *used area* corresponds to the area burned by the fire (hashed) as mapped by MTBS^22^. The *available area* corresponds to the burned area (i.e., used) plus an outside buffer. The buffer width is defined so that the buffer has the same area as the fire. We include the burned area into our definition of available because in order to be burned it needed to be available to burn. The target owner is avoided by fire (left panel) if area burned does not intersect with the target owner, but is available within the buffer. A Jacobs value of -1 represents perfect avoidance when 100% of the target owner is available but unburned. On the opposite end of the Jacobs' range is preference with Jacobs' = 1 (right panel). A fire shows perfect preference for the target owner when area burned is completely contained within the used area. Indifference (Jacobs' = 0; middle panel) happens when the proportion of the fire in the used and the available areas are the same. Figure produced with ArcGIS Pro 2.4.1 (www.esri.com).

For each fire footprint, we defined the *area used* as the area burned by the fire. We defined the *area available* to burn as the area used + the area of a variable width buffer outside the fire footprint (Fig. 4). We used variable buffer widths so that the external buffer had the same size as the burned area ensuring that burned and unburned had equal sampling. We included the area used (i.e., burned) in the availability to burn because logically, in order to be used it needed to be available. When the available area was outside the study area (and thus ownership could not be assigned), the observation was excluded from the analysis. This resulted in 1542 individual fire footprints for analysis.

We calculated the Jacobs' selectivity index^28^ for each fire footprint as follows:

$$D_{ij} = { }\frac{{o_{i} - \hat{\pi }_{i} }}{{o_{i} + \hat{\pi }_{i} - 2o_{i} \hat{\pi }_{i} }}$$

where $o_{i}$ is the proportion of used ownership *i* and $\hat{\pi }_{i}$ is the proportion of available area in ownership *i*. The index ranges between − 1, when a fire exhibited extreme avoidance of ownership *i,* and 1, when the ownership was extremely preferred. Jacobs' values around zero indicated that fire *j* was indifferent to ownership *i*. We plotted the distribution of Jacobs' index per ownership and broad vegetation type, including the median value and corresponding 95% confidence interval, and first and third quantiles.

Ownership is associated with a specific individual footprint if the ownership was available to burn by the footprint; each footprint is one observation and the collection of all the footprints available to a given ownership is used to determine whether there is a pattern of preferential burning for that ownership. As such, different ownerships will have different sample sizes resulting from patterns of fire ignition and spread in relation to the spatial distribution of ownership categories. A fire footprint can interact with just one or multiple ownerships. The same fire footprint can have different selectivity patterns, preferring specific ownerships and avoiding or being indifferent to others.

To determine whether selectivity patterns differed by ownership, we used a nonparametric sign test using the R package *phuassess*^32^. The sign test determines whether a given ownership was preferred, avoided, or ignored by wildfire^33,34^. For each ownership the sign-test statistic is calculated as:

$$t_{j} = {\text{max}}\left( {n_{j}^{ + } , n_{j} - n_{j}^{ + } } \right)$$

where $$n_{j}^{ + }$$ is the number of fires with proportion of use greater than availability and $$n_{j}$$ is the number of fires for which the ownership is available. The *P* value for each sign-test statistic is derived from a binomial distribution under the proportional use hypothesis set with parameter $$n_{j}$$ and 0.5. The resulting *P*-values are combined in a permutation procedure to derive an overall test statistic (across all ownerships) whose significance can be determined by permuting sample observations^33,34^. The overall test statistic corresponds to the minimum *P*-value calculated for each ownership. Its significance (overall *P*-value) was calculated using 50,000 permutations with an alpha of 0.05. If the overall *P*-value allows for the rejection of proportional use, individual ownerships are then grouped as preferred or avoided. This is done by comparing individual *P* values with the alpha parameter (0.05) and the fraction of fires with proportion of use greater than availability ($$f_{j} = {\raise0.7ex\hbox{${n_{j}^{ + } }$} \!\mathord{\left/ {\vphantom {{n_{j}^{ + } } {n_{j} }}}\right.\kern-\nulldelimiterspace} \!\lower0.7ex\hbox{${n_{j} }$}}$$). If the *P* value is > 0.05 then the ownership is proportionally used. If the *P *value ≤ 0.05, then preferred ownerships are those where $$f_{j}$$ ≥ 0.5 and avoided ownerships are those where $$f_{j}$$ < 0.5^33^. All statistical analyses were conducted in R^35^, map figures were produced with ArcMap 10.6.1^36^ and violin plot figures were produced with MatLAB^37^.

## Results

### Ownership and vegetation type classification of fire footprints

The selectivity patterns presented below were based on a sample of footprints that varied between a minimum of 115 for tribal and a maximum of 864 for private non-industrial (Table 1). The large number of footprints that burned on federal lands, FS-matrix, FS-wilderness, and BLM account for 63% of the total burned area in the study area. Together these ownerships account for 60% of the land base (Table 1).

**Table 1:** Percentage of study area and area burned, and fire sample size in forest, grass/shrub and mixed vegetation types, per ownership category.

Ownership	% of study area	% of total area burned	Number of fires by vegetation type			
			Forest	Grass/Shrub	Mixed	Total
FS-matrix	18	15	303	90	52	445
FS-wilderness	14	18	236	39	30	305
BLM	28	30	108	614	46	768
Tribal	7	8	22	80	13	115
State/Washington	5	4	49	307	25	381
State/Oregon	2	1	37	158	16	211
Private non-industrial	16	13	138	661	65	864
Private industrial	2	2	118	113	18	249
Other federal	4	5	38	166	12	216
Other	4	4	22	169	17	208

A fire is associated with a given ownership if the area used, available or both overlap with the ownership. One fire event can be associated with multiple ownerships.

The majority of fires with private non-industrial lands were classified as grass/shrub vegetation (76%). Similarly, BLM and tribal lands were predominantly associated with grass/shrub fires, 80% and 70% respectively. State/Oregon and State/Washington lands were primarily associated with grass/shrub fire events, corresponding to 75% and 80% of all fires that interacted with these ownerships. By contrast, 68% of FS-matrix and 77% of FS-wilderness fires burned in forest. Private industrial lands were associated with a similar number of forest versus grass/shrub fires (47% and 45% respectively). The percentage of unburnable in the available area varied from 1% in private industrial fires to 25% in private non-industrial (Supplemental Material, Table S1).

### Patterns of preference, avoidance, and indifference by ownership category for individual fire footprints

**Figure 5:**
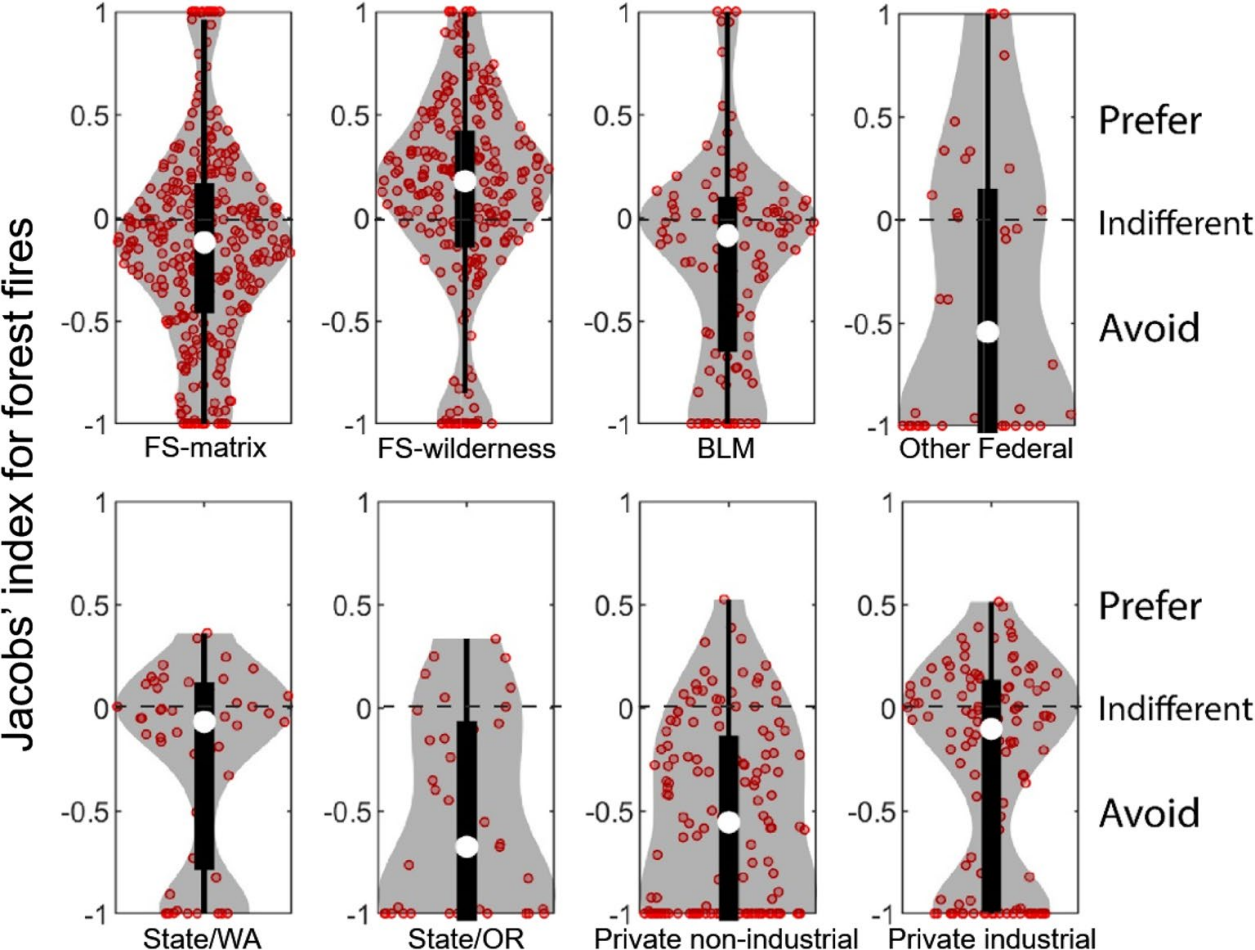
Violin plots showing the distribution of Jacobs' selectivity index values by ownership for forest fires (red circles). Jacobs’ = 1 indicates perfect preference, Jacobs’ =  −1 indicates perfect avoidance and Jacobs’ = 0 represents indifference. Ownerships associated with fewer than 25 observations are not shown (Other and Tribal). White dot represents the median value, thick black lines represent the interquartile range and the thin vertical lines show the 95% confidence interval for the median. Grey shaded area shows a rotated kernel density plot on each side. A horizontal jitter factor was applied to distribute data points horizontally so that similar values did not overlap on the same point. Figure produced with MatLAB 2019a (www.mathworks.com).

Overall, Jacobs' index values of individual footprints burning in forest showed avoidance for most ownerships except FS-wilderness, where fire footprints burned more than would be expected given this ownership’s availability (Fig. 5). Comparison of median values indicates that State/Oregon lands were the least likely to burn given their availability, followed by private non-industrial and other federal. FS-matrix and private industrial, State/Washington and BLM showed weaker patterns of avoidance, with medians closer to indifference (Jacobs’ = 0). Variability of Jacobs' values (shapes of the violin plots) for fires within the forest vegetation type also differed among ownerships. State/Washington, State/Oregon, private non-industrial, and private industrial had no observations with Jacobs’ > 0.5, i.e., the violins plots were truncated due to low levels of disproportionate burning. While the median Jacobs’ index values for FS-matrix and BLM lands indicated avoidance, a small number of individual fires showed a strong preference for these federal ownerships, unlike the private and state lands.

**Figure 6:**
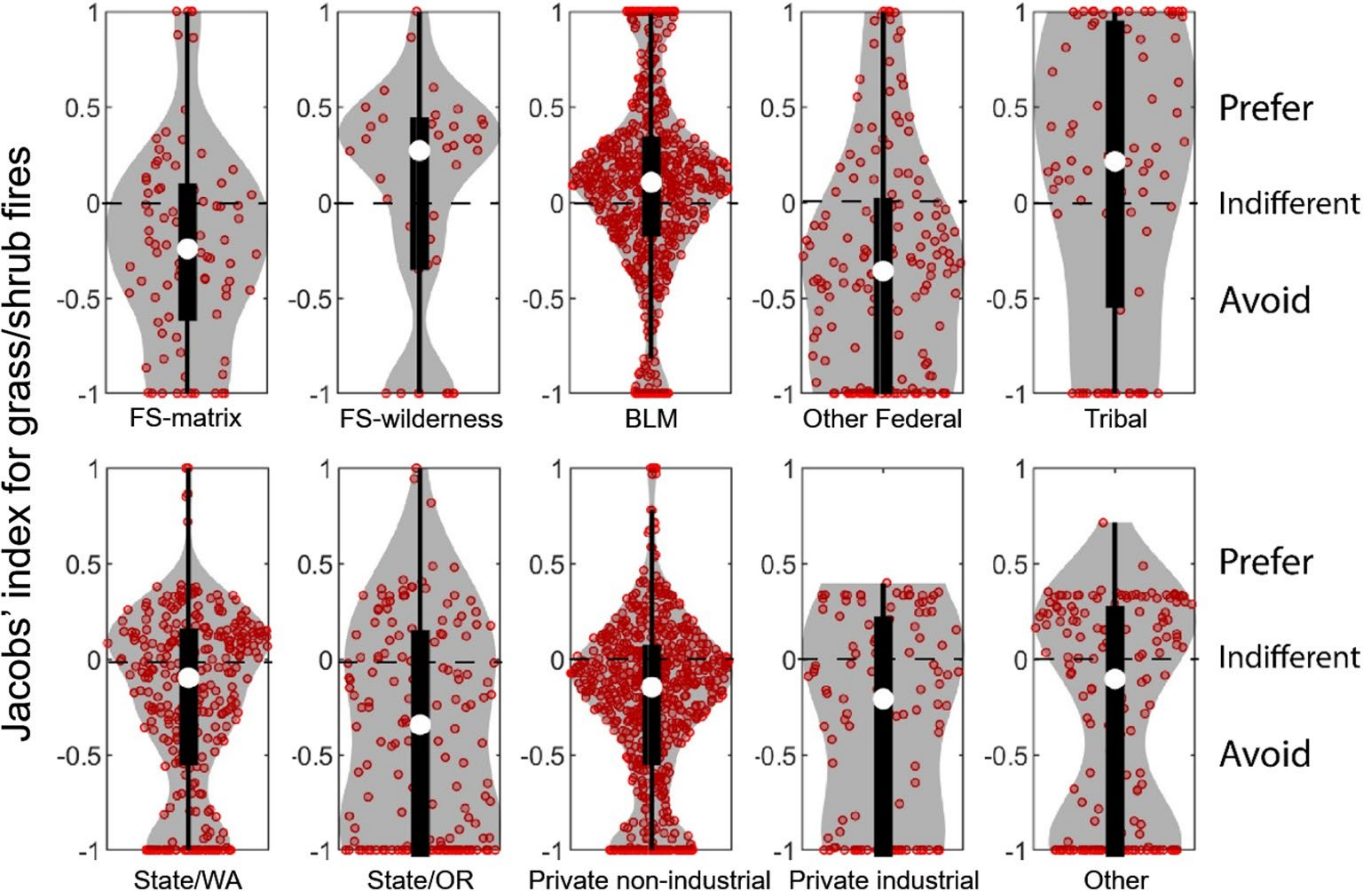
Violin plots showing the distribution of Jacobs' selectivity index for each ownership for grass/shrub fires (red circles). Jacobs’ = 1 indicates perfect preference, Jacobs’ = − 1 indicates perfect avoidance and Jacobs’ = 0 represents indifference. White dot represents the median value, thick black lines represent the interquartile range and the thin vertical lines show the 95% confidence interval for the median. The grey shaded area shows a rotated kernel density plot on each side. A horizontal jitter factor was applied to distribute data points horizontally so that similar values did not overlap on the same point. Figure produced with MatLAB 2019a (www.mathworks.com).

For grass/shrub fires, overall, Jacobs' index values across ownerships showed more variability than for forest fires, with all ownerships having events that showed either perfect preference (Jacobs’ = 1) or perfect avoidance (Jacobs’ = − 1) with the exception of private industrial and other (Fig. 6). Despite greater variability than with forest fires, most ownerships had more grass/shrub fires showing avidance than preference, as indicated by the shape of the violin plot with wider bases than tops. The exception is FS-wilderness, BLM and tribal land where grass/shrub fires with Jacobs’ = 1 were more frequent than observations with Jacobs’ = − 1. Analysis of medians of grass/shrub fires shows that BLM and tribal were generally preferred by fire. FS-matrix, State/Washington, State/Oregon, private non-industrial and private industrial were avoided by fire. However, this avoidance is weaker than observed for forest fires, i.e., higher median Jacobs’ values for fire footprints in grass/shrub than in forest.

**Figure 7:**
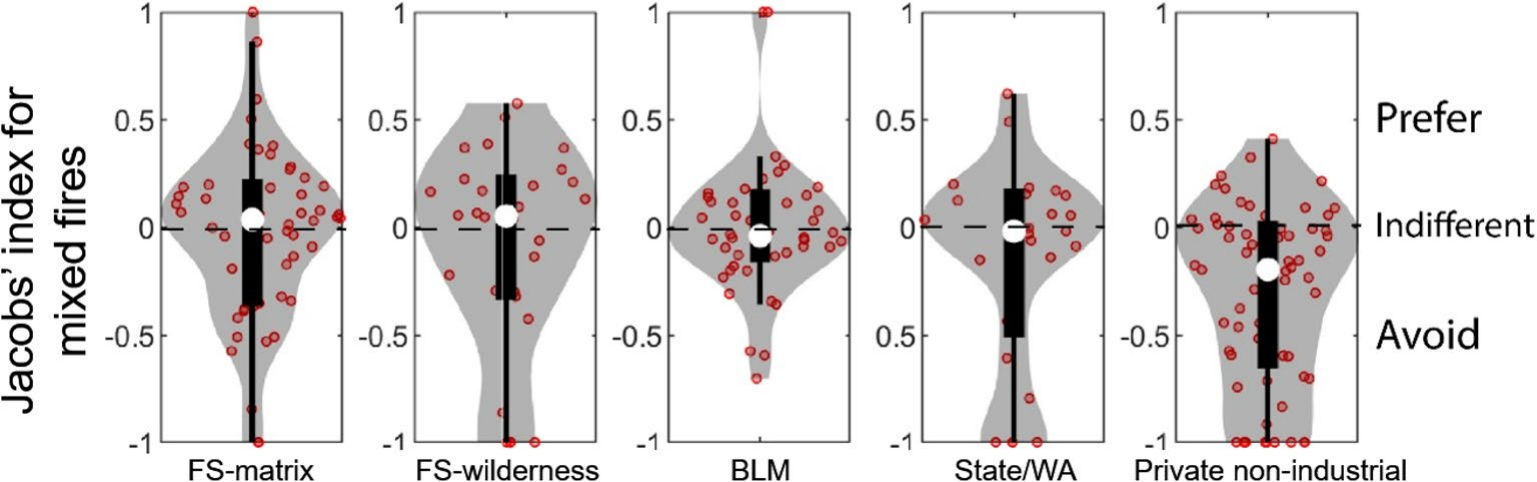
Violin plots showing the distribution of Jacobs' selectivity index for each ownership for mixed vegetation fires (red circles). Jacobs’ = 1 indicates perfect preference, Jacobs’ = − 1 indicates perfect avoidance and Jacobs’ = 0 represents indifference. Ownerships associated with fewer than 25 observations are not shown. The white dot represents the median value, thick black lines represent the interquartile range, and the thin vertical lines show the 95% confidence interval for the median. The grey shaded area shows a rotated kernel density plot on each side. A horizontal jitter factor was applied to distribute data points horizontally so that similar values did not overlap on the same point. Figure produced with MatLAB 2019a (www.mathworks.com).

Fire footprints burning in mixed vegetation types were fewer and occurred on only five ownerships (Fig. 7). Comparison of medians showed that private non-industrial burned less than available on mixed vegetation, whereas fires on FS-matrix, FS-wilderness, BLM, and State/Washington lands burned proportional to availability.

### Patterns of preference, avoidance, and indifference by ownership category at the landscape scale

Nonparametric testing on the proportions burned versus available by ownership showed that FS-wilderness was consistently preferred by fire (Table 2), i.e., FS-wilderness disproportionally burned more than available for both forest and grass/shrub fires. For mixed vegetation fires, FS-wilderness burned proportional to availability. FS-matrix lands were avoided for both forests and grass/shrub fires, and burned proportional to availability for mixed fires. Private industrial lands had similar patterns of preference/avoidance as FS-matrix lands, and the two ownerships ranked consecutively in terms of disproportionate use (Table 2).

**Table 2:** Preference ranking in which the ordering of the ownership column represents the ranking of different land ownerships by decreasing preference, i.e. from preference to avoidance.

Vegetation type	Ownership	F statistic	Overall *P* value	Decision
Forest	FS-wilderness	0.6653	< 0.001	Preferred
	Tribal	0.5909	0.5235	Proportionally used
	Other federal	0.3684	0.1444	Proportionally used
	BLM	0.3704	0.0091	Avoided
	Private industrial	0.3644	0.0041	Avoided
	FS-matrix	0.3531	< 0.001	Avoided
	State/Washington	0.3469	0.0444	Avoided
	State/Oregon	0.1892	< 0.001	Avoided
	Private non-industrial	0.1594	< 0.001	Avoided
	Other	0.0909	< 0.001	Avoided
Grass/shrub	Tribal	0.6625	0.0049	Preferred
	BLM	0.627	< 0.001	Preferred
	FS-wilderness	0.641	0.1081	Proportionally used
	Other	0.4675	0.4419	Proportionally used
	State/Washington	0.4169	0.0042	Avoided
	Private industrial	0.3451	0.0013	Avoided
	FS-matrix	0.300	< 0.001	Avoided
	Private non-industrial	0.2995	< 0.001	Avoided
	State/Oregon	0.2975	< 0.001	Avoided
	Other federal	0.2349	< 0.001	Avoided
Mixed	FS-wilderness	0.5667	0.5847	Proportionally used
	FS-matrix	0.5385	0.6778	Proportionally used
	State/Washington	0.440	0.69	Proportionally used
	BLM	0.4348	0.4614	Proportionally used
	Private non-industrial	0.2462	< 0.001	Avoided

Ranking is based on nonparametric permutation-based test to assess the statistical significance of H0 of proportional ownership use for each fire type (forest fires, grass/shrub and mixed fires).

Within grass/shrub BLM lands were preferred, but within forest fires showed patterns of avoidance. When mixed fires were considered, BLM lands burned proportional to availability. Tribal lands were preferred when considering grass/shrub fires with the highest preference among all ownerships but were proportionally burned by forest fires.

Taken as whole, fires in all vegetation types avoided private non-industrial lands. In other words, fires always burned less than available for this ownership regardless of whether it was a forest, grass/shrub, or mixed fire. Forest fires and grass/shrub fires consistently burned less than expected within lands managed by State/Oregon and State/Washington.

## Discussion

Our results support the hypothesis that lands in Oregon and Washington where homes and timber are concentrated (private non-industrial, private industrial, FS-matrix and state lands) burned less than expected based on their availability to fire. This finding was consistent across vegetation types although avoidance of these same ownerships when considering grass/shrub fires was weaker than for forest fires. We expected private non-industrial lands to be less preferred by fire (of any type) than any of the other ownerships due to the presence of people and structures. Our results support that hypothesis except for grass/shrub fires where lands managed by the state of Oregon were the most avoided, followed by private non-industrial. We hypothesized and found that FS-wilderness areas would burn more than expected on all vegetation types. Tribal and BLM lands also burned more than expected, but only when grass/shrub fires were considered.

Our analysis describes patterns of disproportional burning once a fire encounters a parcel of specific ownership. Limitations of this study include errors on ownership assignment and mapping, as well omission and commission errors associated with MTBS fire perimeters^22^. The MTBS fire atlas covers 27 years of fire, and it's possible that for specific events, ownerships at the time of the fire are different from what is on our ownership map. Similarly, unburned islands within the MTBS fire perimeters and burned areas that are missed in MTBS mapping products are additional data limitations. We posit that these limitations do not detract from our results given the large sample size used to determine disproportionate burning for each ownership, i.e., between a minimum of 115 and a maximum of 864. Errors associated with ownership and burned area mapping would need to consistently affect one specific ownership across multiple fires, which is unlikely.

Potential causes of the patterns observed in this study, i.e., why some ownerships are preferred while others burn less than expected, and why many fires ignore ownership lines altogether, are complex and more difficult to disentangle. Quantifying drivers of observed disproportionate burning by ownership deserves additional study and is beyond the scope of this investigation, although the results support some interpretations based on distinct land management policies in these ownership categories.

For instance, wilderness areas within national forests are places where fire management plans allow wildfire to burn to meet resource objectives, including restoration of fire resilient, pre-European settlement conditions^9^. The policy has had a significant impact on area burned in the wilderness to the point that the current rate of burning is close to pre-settlement conditions in several wilderness areas^38^. Reilly et al.^39^ showed that recent fire frequencies in high elevation forests in the eastern Cascades of Oregon and Washington, where many wilderness areas are located, are similar to historical frequencies. Johnston et al.^40^ compared fire extent and severity between roaded and roadless areas on national forests in the western US. Roaded and roadless land designations are generally comparable to the FS-matrix and FS-wilderness ownerships in this study. The authors found that the latter had greater fire extent but found no differences in fire severity between the two management regimes^40^. Our results are in alignment with past work and showed that wilderness areas burned disproportionately more than what was available to fire. These results may reflect an intentional leveraging of wildfire as a restoration tool in these areas. Another concurrent explanation is that the preference of FS-wilderness areas is a consequence of remoteness, inaccessibility to suppression resources, delayed detection and increased fuel loads due to lack of management, which all hinder fire control.

We were less clear on what drives the range of results for disproportionate use of FS-matrix lands. These lands are generally managed for a mix of values, including timber production, wildlife, and recreation, and are protected by a full fire suppression strategy—at least 98% of fires are suppressed before they become large^41^. FS-matrix is predominately comprised of dry-mixed conifer forest that is the primary target for fuels reduction via mechanical treatments or resource objective fire. However, FS-matrix is also a source of exposure to communities and where wildland urban interface and timber values are concentrated, which can also explain the avoidance results^42^.

Despite the avoidance of FS-matrix lands, 38 out of 445 fires showed a strong preference (Jacobs’ > 0.5). Further analysis showed that fire events with a strong preference for FS-matrix land were predominantly in forest fires (28 out of 38) and that all other ownerships associated with these footprints were strongly avoided (mean Jacobs’ = − 0.87, s.d. = 0.21). Visual analysis of fire footprints with Jacobs’ index > 0.5 shows a good spatial alignment between fire boundaries and ownership boundaries (Supplemental Material, Fig. S1). This may reflect full suppression policy by both federal, state, tribal, and local fire agencies to ensure fires stay within FS boundaries. Natural ignitions on FS-matrix can be managed for multiple land management objectives^43^, meaning that an incident can be managed concurrently for resource objectives in one area and aggressive suppression along the boundaries with non-FS ownerships. This could also contribute to the preference for FS-matrix lands shown by these footprints.

The avoidance of all non-FS ownerships is consistent with policy and management goals. Private industrial ownerships have an overarching management goal of timber production and forest protection. Private non-industrial may correspond to small woodlots but are primarily associated with homes and people, thus a top priority for fire suppression and risk reduction (though not with prescribed fire)^2,10^. State agencies have protection responsibilities over private non-industrial and private industrial lands outside cities' fire protection districts. State agencies also have fire protection responsibilities over state trust lands, where timber is the primary highly-valued resource intended to provide revenue for counties and public schools^44^. Protection of homes and timber values from loss to fire is the top priority across both private and state ownership categories. Furthermore, state statutes in both Oregon and Washington declare wildfire to be a public nuisance and enforce a full suppression policy to reduce acres burned. While some scientists have advocated for greater use of prescribed fire and management of wildfire for resource objectives in dry forests^9,44,45^, the option to manage natural ignitions for environmental benefits is not the current policy for the state of Washington or Oregon.

Tribal and BLM lands were preferred in grass/shrub fires. On tribal lands, it is possible that the preference by fire is evidence of efforts to increase the practice of cultural burning, which was severely limited by colonization and associated fire management^46^. BLM lands were burned by grass/shrub fires more than they were available, which we attribute to the spatial extent and remoteness of this ownership category combined with fast spread rates associated with grass/shrub fuels^47^. Fast-spreading fires, delayed detection, and lack of resources in remote areas where these fires occur can contribute to the observed patterns of fire preference on BLM.

Our analysis supports the common assertion that in most cases, fire spread is not influenced by ownership boundaries, as evidenced by the significant variability in Jacobs' values and the shape of the violin plots where the widest portion is in the indifference region (middle, Jacobs’ = 0). Patterns of preference, avoidance, or indifference for individual fires also likely reflect the effects of fire weather during the event^3^. Weather moderates, and in many cases, overrides the effects of fuel availability and suppression efforts on the ability to control fire spread^48^. Footprints that were indifferent to ownership are likely the result of fire weather conditions that limit the ability of firefighters to control fire spread. Previous work to correlate fire selectivity to land cover types used fire size as a proxy for weather conditions and found that as fire size increased, patterns of preference and avoidance for specific land cover types trended towards indifference^31^.

The relationship between fire and ownership has received little attention in terms of empirical studies, although wildfire simulation studies have studied cross-boundary fire and risk transmission. Zald and Dunn^3^ analyzed fire severity in different portions of the 2013 Douglas Complex fire that burned 19,000 ha of checkerboard pattern of private industrial and BLM lands. Their results show that daily fire weather was the most important predictor of severity followed by stand age and ownership. Furthermore, severity was higher on private industrial lands, dominated by younger plantations than on BLM forested lands, where there is a much greater proportion of older forest. Using simulated fires over the course of 50 years, Charnley et al.^10^ investigated patterns of disproportionate burning of high-severity fire on FS, State, and private industrial lands in southern Oregon. They found that private industrial burned disproportionally more than expected where state and FS lands burned less than expected. Ager et al.^4^ using simulated fires showed that the shape and size of parcels affected cross-boundary fire transmission.

Understanding the complexities of fire selectivity on the overall management of the wildland fire system can inform various initiatives aimed at coordinating fire and fuel management across land ownership^17,49^. Our results contribute to a shared understanding of how diverse land management strategies play out on the landscape. This can contribute to better communication among landowners and with the public, and build the trust necessary to work across ownerships^16^.

There is a wide spectrum of suppression policies that range from fortress protection^10^ to living with wildfire with expanded use of resource objective wildfire to improve fire resiliency on fire-excluded landscapes^9^. However, our results suggest that despite diverse cross-boundary policies, landscape outcomes are similar across ownerships, with forest fires burning less than what would be expected across all ownerships except FS-wilderness. While it is impossible to determine manager's intent from the analysis of fire footprints, our results suggest that the use of resource objective wildfire is limited to wilderness areas^38,50–52^. In these landscapes, fortress protection is the common approach across all lands and counterproductive to federal forest restoration policies and the broad goal of expanding the footprint of resource objective fire^15^.

Another key finding is that despite patterns of selectivity, when the fire record is taken as a whole, many fires are indifferent to ownership. If we take ownership as a proxy for suppression policy this suggests that in many cases suppression will not be capable of altering fire spread, limiting the efficacy of the fortress protection approach. Recognizing and communicating the limitations of suppression efforts is fundamental to set reasonable expectations with the public and landowners.

Creating conditions for fire to occur safely via treatments that improve forest resilience requires educating communities on the role of fire in fire-adapted landscapes. At the same time, it is important to recognize that in some places living with fire means protection is the only strategy compatible with forest and fire management goals. In multi-ownership landscapes, informed discourse on shared wildfire exposure is critical to reduce conflict among state, federal and private land ownerships and support effective cross-boundary initiatives to expand the pace and scale of forest restoration programs that are designed in alignment with fire management goals.

## Supplementary Information

Supplementary Information 1.
