# Supplementary material for: Effects of ownership patterns on cross-boundary wildfires
Source: Sci Rep. 2021 Sep 29;11:19319. doi: 10.1038/s41598-021-98730-1 (PMC8481543; doi:10.1038/s41598-021-98730-1)
Supplement: Supplementary file 1 — Supplementary Information 1. [file 41598_2021_98730_MOESM1_ESM.docx]

Supplemental material for

Effects of ownership patterns on cross-boundary wildfires

Ana M.G. Barros^1*^, Michelle A. Day^2^, Thomas A. Spies^3^, Alan A. Ager^2^

*Corresponding author

^1^Oregon State University, College of Forestry, Corvallis, OR, USA

^2^USDA Forest Service, Rocky Mountain Research Station, Missoula Fire Sciences Laboratory, Missoula, MT, USA

^3^USDA Forest Service, Pacific Northwest Research Station, Corvallis, OR, USA

**Table S1.** Percentage of available are that is classified as nonburnable. See figure 4 for a description of used and available areas for individual fire events.

| Ownership | Percent of available area that is nonburnable |
| --- | --- |
| FS-matrix | 2% |
| FS-wilderness | 2% |
| BLM | 4% |
| State/Oregon | 6% |
| Other fed | 14% |
| Tribal | 4% |
| Private non-industrial | 25% |
| State/Washington | 14% |
| Private industrial | 1% |


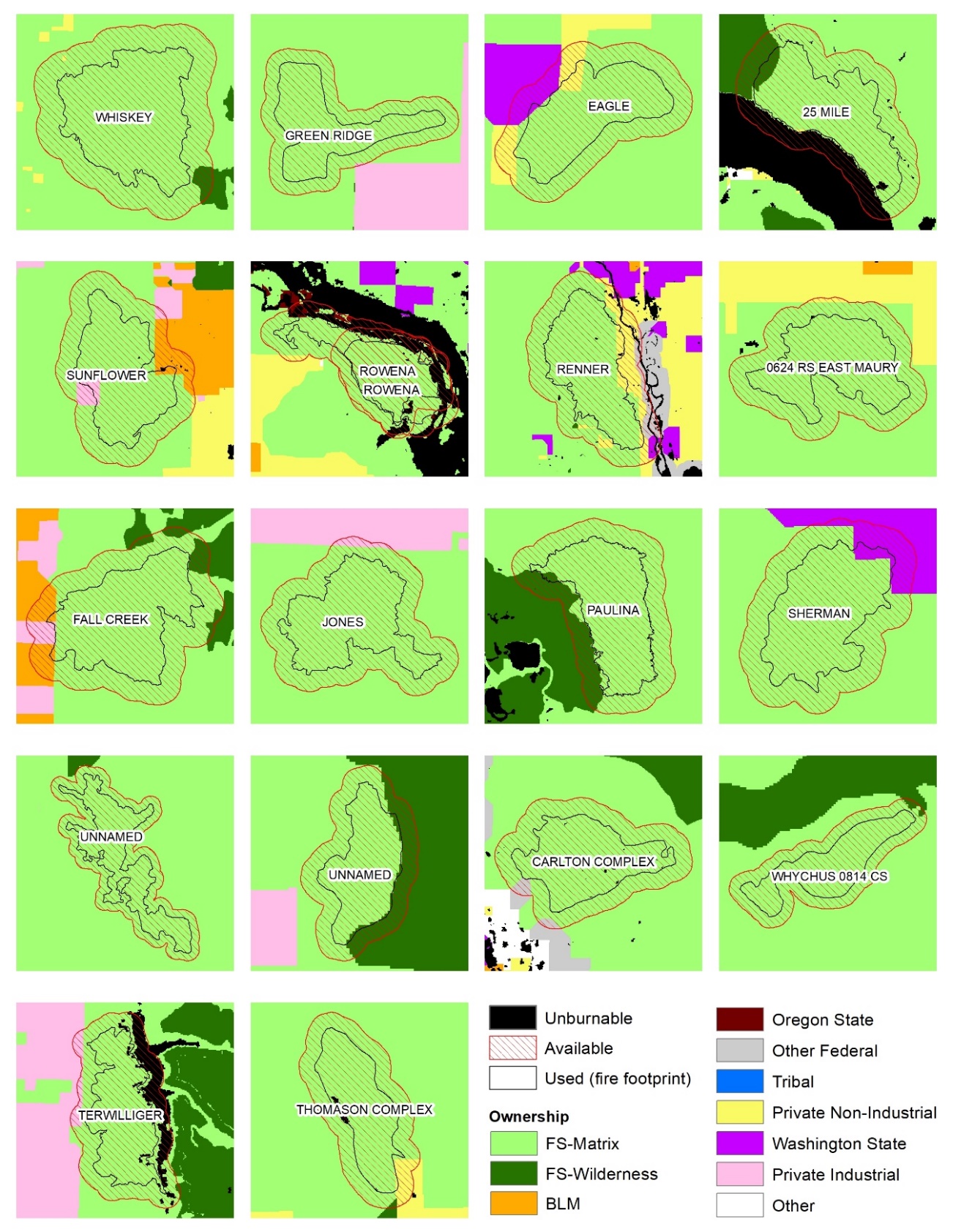


**Figure S1**. Fire footprints with preference for FS-matrix lands (Jacobs' selectivity index >0.5) and corresponding available areas overlayed. Unburnable areas (black) were superimposed on ownership classes.


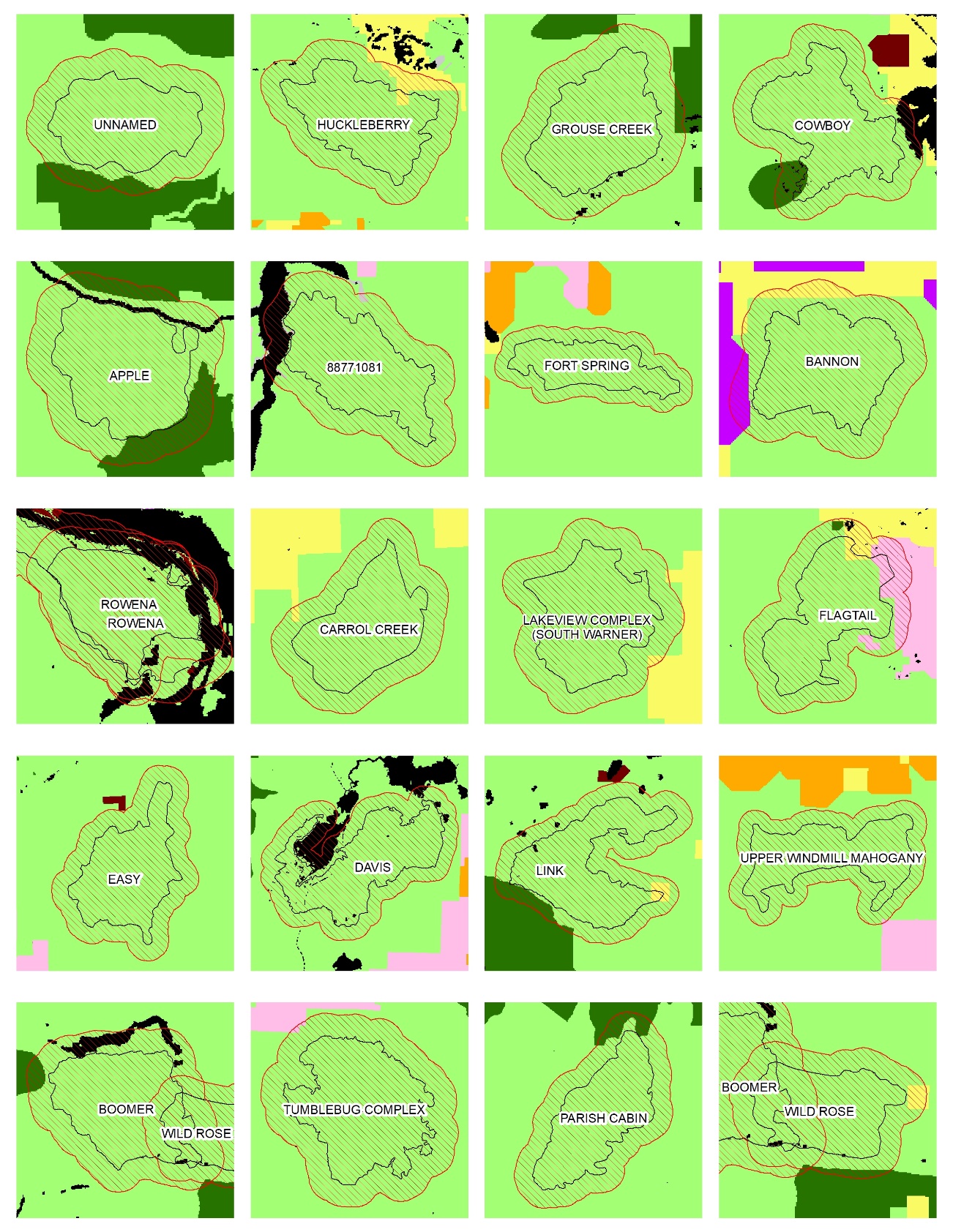


**Figure S1 (continued).** Fire footprints with preference for FS-matrix lands (Jacobs' selectivity index >0.5) and corresponding available areas overlayed. Unburnable areas (black) were superimposed on ownership classes.
